# Morphological, physio-biochemical and nutritional status as potential markers for grafting compatibility in Kalamata olive cultivar

**DOI:** 10.1186/s12870-023-04346-0

**Published:** 2023-06-22

**Authors:** Ahmed AbdelHady Rashedy, Hamed Hosni Hamed

**Affiliations:** Pomology department, Faculty of Agriculture, Cairo University, Giza, Egypt

**Keywords:** Olea europaea, ABA, GA, IAA, Enzyme, Mineral, Sugar, Phenol, Rootstock, Scion

## Abstract

**Background:**

Choosing the most compatible and desirable rootstock for Kalamata olive cultivar is an important decision due to the longevity of the orchard and the difficulty rooting of Kalamata cuttings. Therefore, the goal of this study was to examine the morphological, physio-biochemical, and nutritional status as ppotential markers for grafting compatibility between Kalamata olive cultivar and three olive rootstocks (Coratina, Picual, Manzanillo) during two seasons (2020–2021) as well as follow up physio-bichemical and nutritional status of one-year-old Kalamata plants (2022).

**Results:**

The results indicated that, Picual rootstock recorded the highest significant grafting success which was associated with increasing number of leaves, leaf area and SPAD value in Kalamata scions by 22.15%, 36.86% and 14.64% compared to Manzanillo rootstock as mean of both seasons, respectively. While, Manzanillo rootstock recorded the highest significant activity for peroxidase and catalase by 51.41% and 60.1% at grafting union compared to Picual rootstock. Moreover, Picual rootstock for Kalamata scions had the highest acid invertase and sucrose synthase activities by 67.23% and 57.94% compared to Manzanillo rootstock. Furthermore, Picual rootstock recorded the highest significant Gibberellic acid by 52.8% and 18.6% compared to Coratina and Manzanillo rootstocks. Meanwhile, Picual rootstock recorded the lowest significant Abscisic acid by 68.17% and 63.15% as well as the lowest total phenols by 14.36% and 23.47% compared to Coratina and Manzanillo rootstocks.

**Conclusions:**

This study sheds light for the importance of choosing the suitable rootstock for Kalamata cultivar. Also, sucrose synthase and acid invertase may have a novel role in determining grafting compatibility in olives. Increasing growth promoters (Gibberellic, Nitrogen) and decreasing both growth inhibitors (Abscisic, phenols) and oxidative enzyme (catalase, peroxidase) required for better graft compatibility.

## Background

Olives (Olea europaea L.) are among the oldest cultivated fruit trees with the most important socially and economically in the Mediterranean region. Global production of table olives for the year 2022/2023 was estimated at about 3100.000 tons, of which Egypt’s production is 19.4% [1]. In Egypt, olive trees consider a strategic crop due to their high adaptability to drought, salinity and hot summer temperature [2, 3]. Kalamata olive cultivar is a dual-purpose cultivar (table olives and olive oil) and is considered one of the most popular Greek varieties worldwide, with black table olives desirable for customers. Unfortunately, Kalamata leaf cuttings have been described as recalcitrant and hard-to-root cultivar which negatively affects the production of commercial nurseries and orchards [4, 5].

Therefore, the grafting technique is the cheapest alternative to commercial propagation and has additional advantages in rootstock. Choosing the appropriate rootstock for Kalamata cultivar has not received much attention from the scientific community. Rootstocks affected plant vigor, nutritional status and overcoming biotic and abiotic stress [6–8].

Previous studies indicated that, Arbequina cv. followed by FS-17 cv. recorded the highest grafting success and number of leaves, whereas Kalamata cv. had the lowest grafting success and number of leaves when grafted onto wild olive rootstock [9]. Grafting Ayvalik and Domat olive cultivars onto Gemlik rootstock showed slowly cambium cells formation, which contains greater concentrations of phenolic compounds (TP) than Sari Ulak and Memecik cultivars [10]. In grapevine, lower TP content and peroxidase activity in the grafting zone were coincided with a higher grafting compatible in Flame Seedless/Paulsen1103 rootstock than Flame Seedless/Freedom rootstock graft combination [11]. Also, Paulsen1103 was considered the most strongest compatible rootstock for *Vitis vinifera* cultivars which was accompanied by the lowest peroxidase activity at the grafting union than Freedom, Salt Creek and 110 Richter rootstocks [12].

More recently, soluble sugar content and SPAD value (chlorophyll indicator) may be used as indices to assess graft compatibility in grafting citrus [13]. Also, Miao et al. [14] found that, sugars had a stimulative effect on graft union development in cucumber/pumpkin grafting combination. Moreover, phytohormones such as auxin (IAA), cytokinin, abscisic acid (ABA) and gibberellin (GA) play an important role in grafting union development [15, 16].

Kalamata cultivar is a Greek cultivar that serves as a scion grafted onto the Italian cultivar Coratina and the Spanish cultivars Manzanillo and Picual [17]. Grafting compatibility is one of the most important criteria in rootstock selection, which has been rarely studied in Kalamata cultivar. Therefore, the aim of this experiment was to choose an appropriate olive rootstock for Kalamata cultivar based on morphological, physio-biochemical and nutritional status indicators.

## Materials and methods

This study was conducted during the period 2020–2022 in the nursery of the Faculty of Agriculture, Cairo University, Egypt (30°01’04"N31°12’30"E). It was aimed to choose the more desirable olive rootstock for Kalamata cultivar. Three olive cultivars namely Coratina, Manzanillo and Picual were used as rootstocks for Kalamata cultivar. The plant sources involved in this study were obtained from the olive collections farm at the Faculty of Agriculture, Cairo University, Egypt.

### Plant preparation

Rootstocks were prepared from semi-hardwood cuttings taken from the mother plants of Coratina, Manzanillo and Picual cultivars and planted into 10 kg planting media one year before each season which considered as rootstocks. Thirty plants with a diameter of 1 cm and a length of 70 cm were chosen from these nurseries after one year.

Table (1) presents soil and water analyzes for this experiment. In February of each season, scion wood was prepared with 10 cm length from Kalamata mother plant with a diameter of 0.6 cm and 4 nodes after which trimmed their leaves and grafted by cleft method into previous prepared rootstocks. Six months later, samples were collected for various parameters in both seasons (2020–2021). In the second year (2021), thirty additional seedlings were grafted to become one-year-old grafted ‘Kalamata’ plants in the following year (2022) to follow up on the performance of Kalamata cultivar one year after grafting.

**Table 1:** Soil and water analysis of the experiment

Sample	pH (1:1)	EC dS/m		Soluble anions (meq-1)					Soluble cations (meq-1)		
				CO3	HCO3	Cl	SO4	Na	K	Ca	Mg
**Water**	7.65	2.84		0.00	5.47	14.89	8.11	17.96	0.26	4.90	5.35
**Soil**	7.78	1.30		0.00	0.60	12.00	0.10	7.50	2.33	11.00	2.60
**Soil (mg) kg-1 soil)**	**N**	**K**		**P**	**Fe**	**Cu**	**Mn**	**Zn**	**B**		
	64.7	103.3		0.266	0.808	0.120	0.042	0.222	0.101		
**Soil physical analysis %**	**Sand %**		**Silt %**		**Clay %**		**Texture class**		**Organic matter (%)**		
	73.32		13.09		13.58		Loamy sand				

## Measurements

### Morphological study

Morphological parameters taking at the end of growth season (first of September). Grafting success was calculated by dividing the number of successful grafted nurseries by the total number of grafted nurseries. The number of leaves were counted and then the leaf area was calculated according to Koubouris et al. [18] from the seventh fully mature leaf from the tip of the stem (leaf area = 0.308 + 0.708* leaf length*leaf width). A Minolta portable chlorophyll meter was used to calorimetrically determine the amount of chlorophyll as a SPAD value [7].

### Biochemical analysis

Samples were taken from the grafting union (1 cm from the top of rootstock and another 1 cm from the base of scion). All biochemical samples taken three months after grafting (May) for current Kalamata grafted plants, while one-year Kalamata samples were taken after 15 months later for the different following biochemical analysis:

### Sugars content

Total sugars (TS) and sucrose were determined in ethanolic tissue extract according to the anthron reagent method, while reducing sugars were determined by the dinitrosalicylic acid method and then samples were read by spectrophotometer at 620 nm and 550 nm, respectively [19, 20].

### Phytohormone determination

From grafting union 0.5 g tissues was taken in liquid nitrogen for Phytohormone determination. Total indoles were determined in methanolic extract using P-dimethyl amino benzaldehyde by spectrophotometer at 530 nm as IAA (mg g^− 1^FW) according to Larsen et al. [21]. After extraction protocol then purification steps and finally quantification of gibberellic acid (GA3,GA4), indole acetic acid (IAA), zeatin (Z) and abscisic acid (ABA) were carried out according to Unyayar et al. [22].

### Total phenol content (TP)

Total phenols were determined in ethanolic extract by Foiln and sodium carbonate (20%) using a spectrophotometer at 765 nm [23]. Then TP were expressed as gallic acid (mg g-^1^).

### Enzyme activity

#### Antioxidant enzymes

From grafting union 0.5 g tissues was taken in liquid nitrogen for peroxidase activity (POX) and catalase activity (CAT) which were determined in the supernatant after extraction protocol (ground, buffering, homogenate solutions) and centrifugation (12,000 for 20 min at 4 °C) according to Bradford [24].

### Sucrose-metabolizing enzymes

Half gram from grafting union tissues was taken in liquid nitrogen for soluble acid invertase (cytosolic) activity (ACI) determination by dinitrosalicylic acid method [25]. While, sucrose synthase (SCS) activity was determined according to Aloni et al. [26].

## Nutritional status

Nutritional status of ‘Kalamata’ scions were determined in dry leaves taken from the 7th full matured leaf from shoot tip at the end of growing season (September). Kjeldahl digestion method was used to determine Nitrogen (N) content of leaf samples [27]. For other nutrients determination, leaf samples were digested in mixture of nitric acid and perchloric acid at 4/1 (V/V) according to Kacar [28]. In the digestion solution Phosphorus (P) was determined by a colorimetric method by means of Barton reagent. Also, inductively coupled was used for determination of K, Na, Ca, Mg, Fe, Cu, Mn, and Zn contents.

## Visual compatibility symptoms

Visual compatibility indices were observed at grafting union in one-year-old kalamata plants grafted onto different rootstock at the end of growing season (September).

## Statistical analysis

This experiment **was** designed as a randomized complete block design (RCBD) with one factor (rootstocks) for arrange treatments which included three replicates for each one. An ANOVA analysis was used for test significance differences between treatments by MSTAT-C statistical package software [29]. Least significant difference (LSD) values were calculated at propability level 0.05 [30]. The data presented are the mean ± standard error of the independent replicates (n = 3).

## Results

### Morphological parameters

**Fig. 1:**
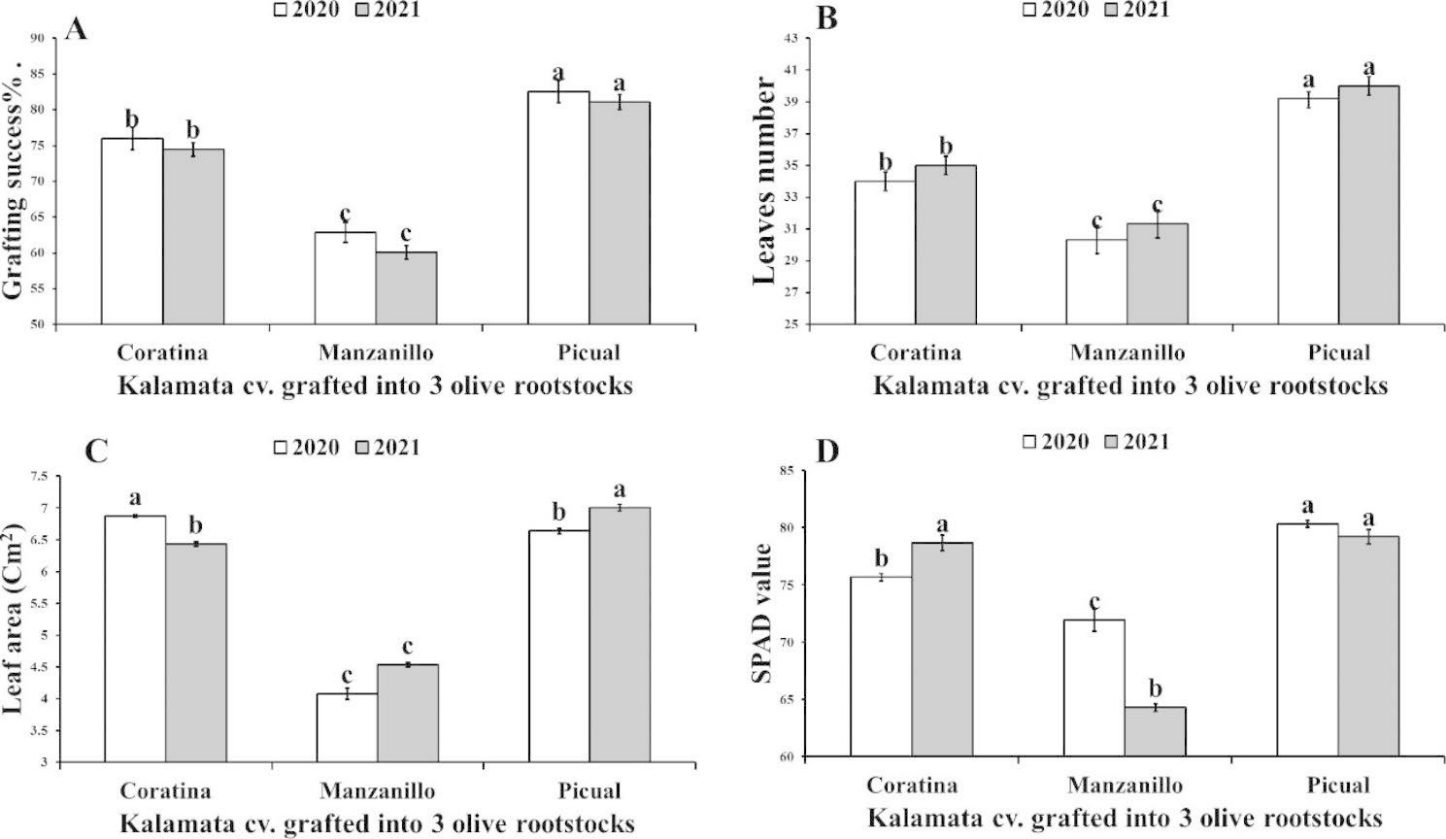
Variation in grafting success % (**A**), leaves number (**B**), Leaf area (**C**) and SPAD values (**D**) of Kalamata scions grafted on three olive rootstocks (Coratina, Manzanillo, Picual) during two seasons (2020–2021). Data are mean ± standard error (n = 3). Different letters between treatments indicate statistically significant differences (*p* < 0.05 level)

It can be noticed that Picual rootstock recorded the highest significant grafting success%, number of leaves, leaf area and SPAD value of Kalamata scions by 23.84% & 25.87%, 22.6% & 21.7%, 38.55% & 35.18% and 10.45% & 18.84% compared to Manzanillo rootstocks in the first and second season, respectively (Fig. 1).

**Fig. 2:**
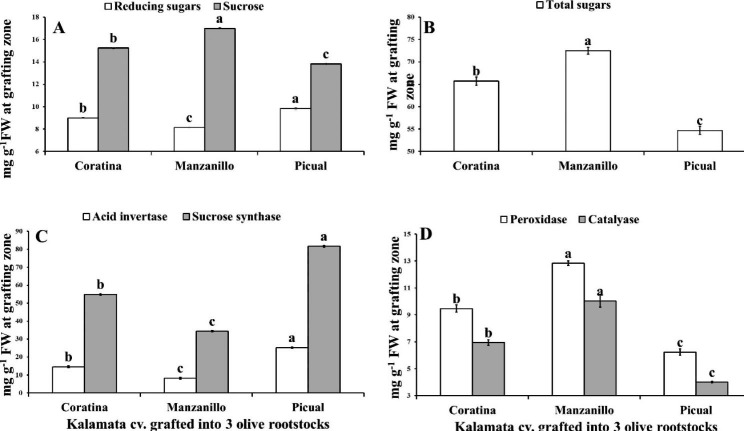
Variation in reducing sugars, sucrose (**A**), total sugars (**B**), acid invertase and sucrose synthase (**C**), peroxidase and catalase (**D**) at grafting union between Kalamata scion grafted onto three olive rootstocks (Coratina, Manzanillo, Picual). Data are mean ± standard error (n = 3). Different letters between treatments indicate significant differences (*p* < 0.05 level)

The data presented in Fig. 2 (A, B) indicated that, Manzanillo rootstock for Kalamata scions recorded the highest significant sucrose and TS by 11.54% & 11.48% and 35.97% & 22.94 at grafting union compared to Coratina and Picual rootstocks, respectively. While, Picual rootstock reordered the highest reducing sugars in grafting union by 33.58% and 8.24% compared to Manzanillo and Coratina as rootstock.

### Enzyme activity

Manzanillo rootstock for Kalamata scions recorded the highest significant activity for POX and CAT by 51.41% and 60.1% at grafting union compared to Picual rootstock which recorded the lowest activity of enzymes (Fig. 2C, D). Also, Picual rootstock for Kalamata scions had the highest ACI and SCS activities by 67.23% and 57.94% at grafting union compared to Manzanillo rootstock which had the lowest activity of enzymes. Moreover, Coratina rootstock recorded moderate activity of enzymes between Picual and Manzanillo rootstocks.

**Fig. 3:**
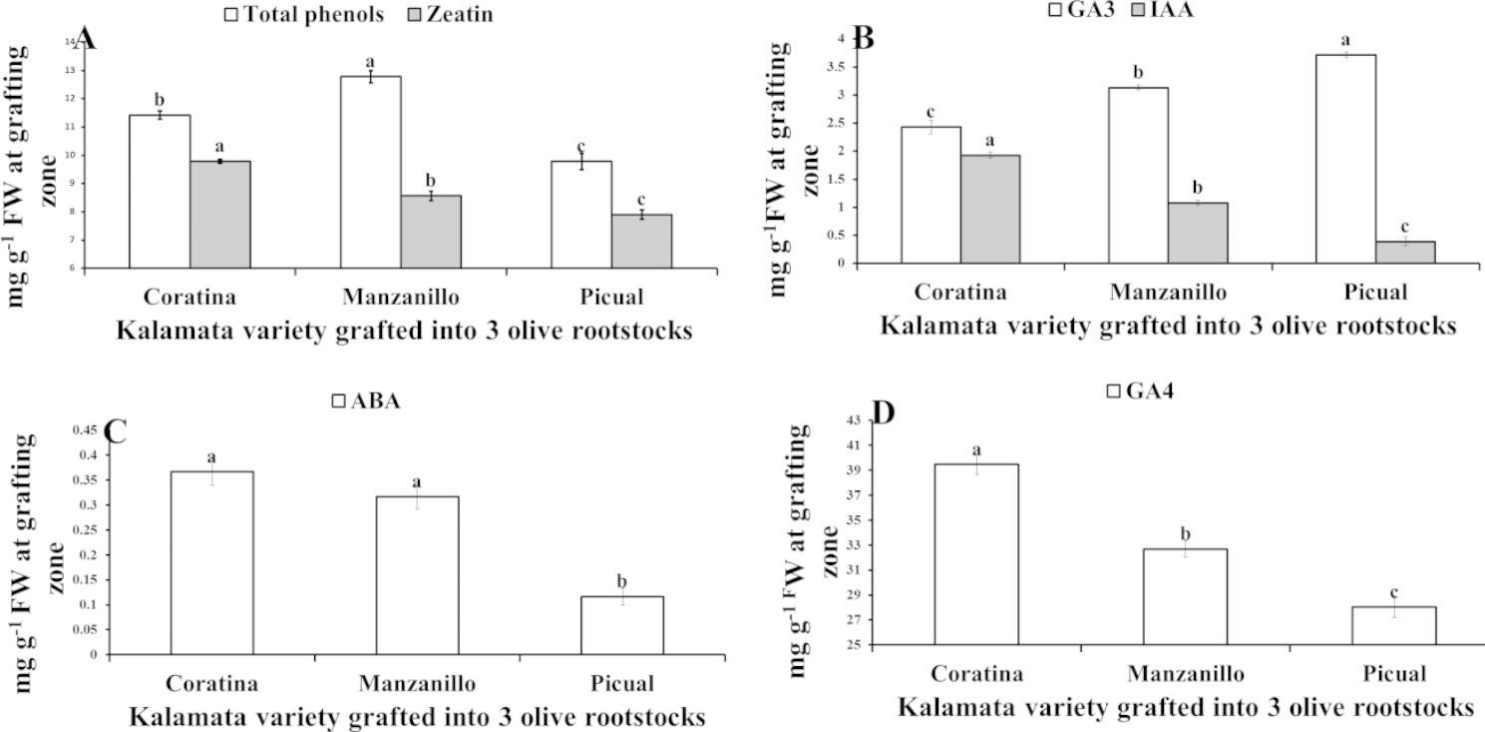
variation in total phenols, zeatin (**A**), GA3, IAA (**B**), ABA (**C**) and GA4 (**D**) at grafting union of Kalamata scion grafted onto three olive rootstocks (Coratina, Manzanillo, Picual). Data are mean ± standard error (n = 3). Different letters between treatments indicate significant differences (*p* < 0.05 level)

Manzanillo rootstock recorded the highest significant TP content, while Picual rootstock recorded the lowest value in the grafting union (Fig. 3A). Meanwhile, Coratina rootstock recorded the highest significant Zeatin content (Fig. 3A). Also, Coratina rootstock recorded the highest significant GA_4_ and IAA content which increased by 20.7% & 78.4% than Manzanillo rootstock and increased by 40.8%&394.1% compared to Picual rootstocks, respectively (Fig. 3B, D). Moreover, Picual rootstock recorded the highest significant GA_3_ by 52.8% and 18.6% compared to Coratina and Manzanillo rootstocks, respectively. By contrast, Picual rootstock recorded the lowest significant ABA by 68.17% and 63.15% compared to Coratina and Manzanillo rootstocks. Also, Picual rootstock recorded the lowest TP by 14.36% compared to Coratina rootstock and lower by 23.47% compared to Manzanillo rootstock (Fig. 3B, C).

**Fig. 4:**
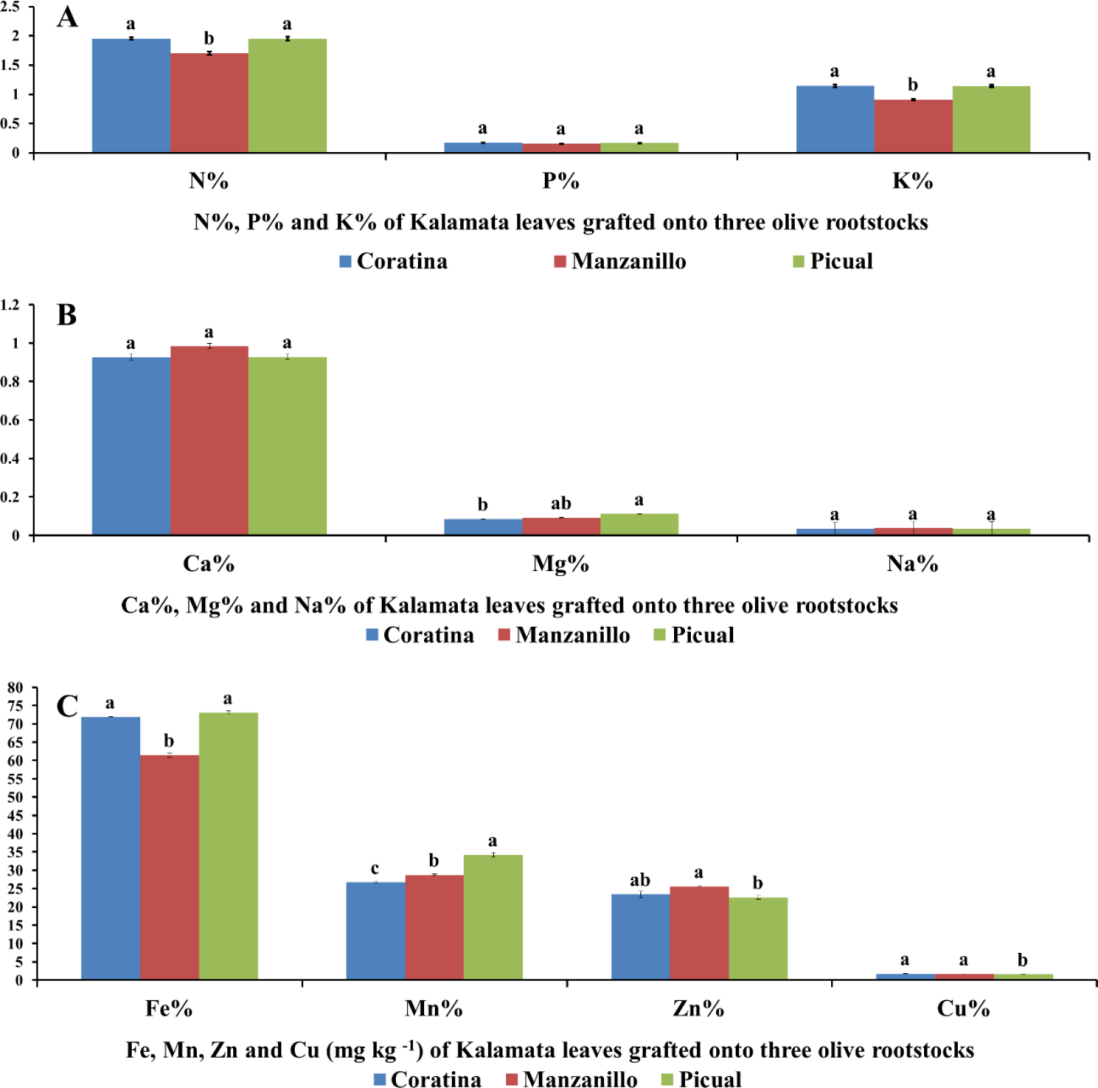
Effect of three olive rootstocks (Coratona, Manzanillo, Picual) on mineral content (**A**) (N, P, K), (**B**) (Ca, Mg, Na), and (**C**) (Fe, Mn, Zn, Cu) of Kalamata scions. Data are mean ± standard error (n = 3). Different letters between treatments indicate significant differences (*p* < 0.05 level)

Coratina and Picual rootstocks recorded the highest significant N by 14.92% and 14.68% and K by 26.04% and 25.60% content in leaves of Kalamata scions compared to Manzanillo rootstock which recorded the lowest significant N, K content(Fig. 4A, B). While there is no effect of rootstocks on Kalamata P% Ca and Na leaves content. While Picual rootstocks accumulate the highest Mg content in Kalamata scion with a significant value compared to Manzanillo (by 23.82%) and 17.50% compared to Coratina and Manzanillor rootstocks, respectively. Moreover, Picual rootstock significantly increased Fe and Mn content in Kalamata scions by 19% and 19.04% compared to Manzanillo rootstocks (Fig. 4C). While Manzanillo rootstock significantly increased Zn and Cu in Kalamata scions compared to Picual rootstock by 11.61% and 9.4%, respectively (Fig. 4C).

### One-year-old Kalamata plants

**Fig. 5:**
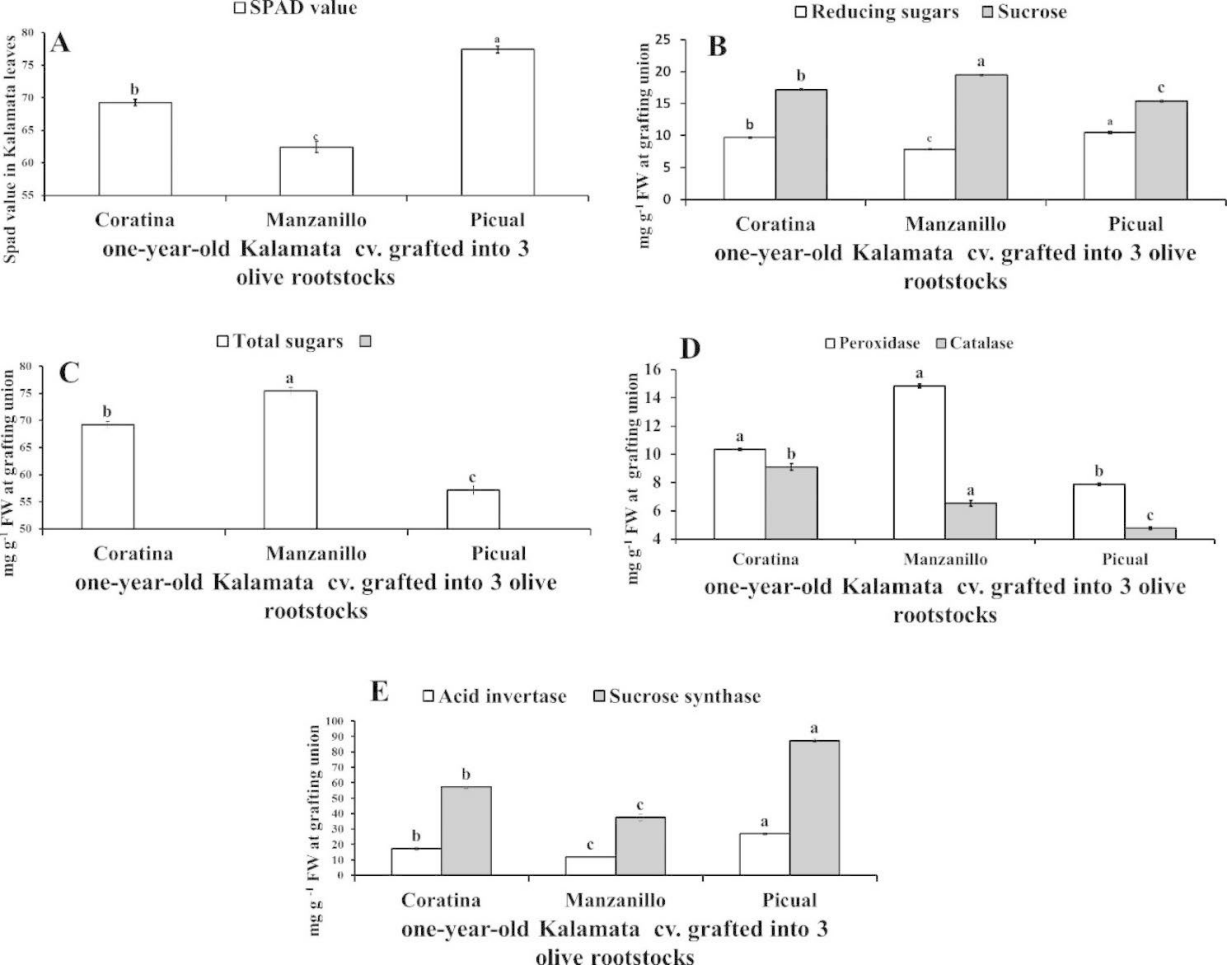
Variation in SPAD value (**A**) sucrose, reducing sugars (**B**), total sugars (**C**), peroxidase, catalase (**D**), acid invertase and sucrose synthase (**E**) in the grafting union of one-year-old Kalamata scion grafted onto three olive rootstocks (Coratona, Manzanillo, Picual). Data are mean ± standard error (n = 3). Different letters between treatments indicate significant differences (*p* < 0.05 level)

In one-year-old Kalamat plants grafted onto three olive cultivars, it can be observed that, Manzanillo rootstock recorded lowest significant SPAD value in Kalamata leaves by 11.76% and 24.05% compared to Coratina and Picual rootstocks (Fig. 5A). While Manzanillo rootstock recorded the highest significant TS and sucrose content by 8.96% & 13.13% and 31.99% & 26.64%, respectively than Coratina and Picual rootstocks (Fig. 5B, C).

In one-year-old Kalamata cultivar, Picual rootstock recorded the lowest significant POX and CAT by 23.84% and 47.62% than Coratina and by 46.86% and 27.06% than Manzanillo rootstocks, respectively (Fig. 5D). While, Picual rootstock recorded the highest significant reducing sugars by 33.59% and 8.25% compared to Coratina and Mnazanillo rootstocks, respectively (Fig. 5B). Also, Picual rootstock recorded higher significant AI, SCS by 55.92% and 52.61% than Coratina rootstock and by126.32% and 132.42% than Manzanillo rootstocks, respectively (Fig. 5E).

**Fig. 6:**
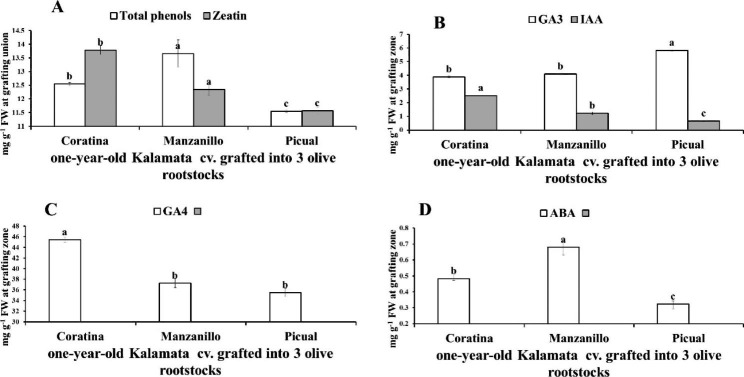
Variation in total phenol, zeatin content (**A**), GA_3_, IAA (**B**), GA_4_ (**C**) and ABA (**D**) in the grafting union of one-year-old Kalamata scion grafted onto three olive rootstocks (Coratona, Manzanillo, Picual). Data are mean ± standard error (n = 3). Different letters between treatments indicate significant differences (*p* < 0.05 level)

In one-year-old Kalamata scion, Coratina rootstock recorded the highest significant Zeatin, GA_4_ and IAA content by 11.66%, 22.01% and 105.62% than Manzanillo and higher by 19.19%, 28.06% and 284.19% compared to Picual rootstock, respectively (Fig. 6A, B, C). While, Picual rootstock recorded the highest significant GA_3_ content by 49.91% and 42.23% than Coratina and Manzanillo rootstock (Fig. 6B). on the contrary, Manzanillo rootstock recorded the highest significant TP and ABA content by 8.66% and 40.70%% than Coratina and higher by 18.27% and 110.33% than Picual rootstocks, respectively (Fig. 6D).

**Fig. 7:**
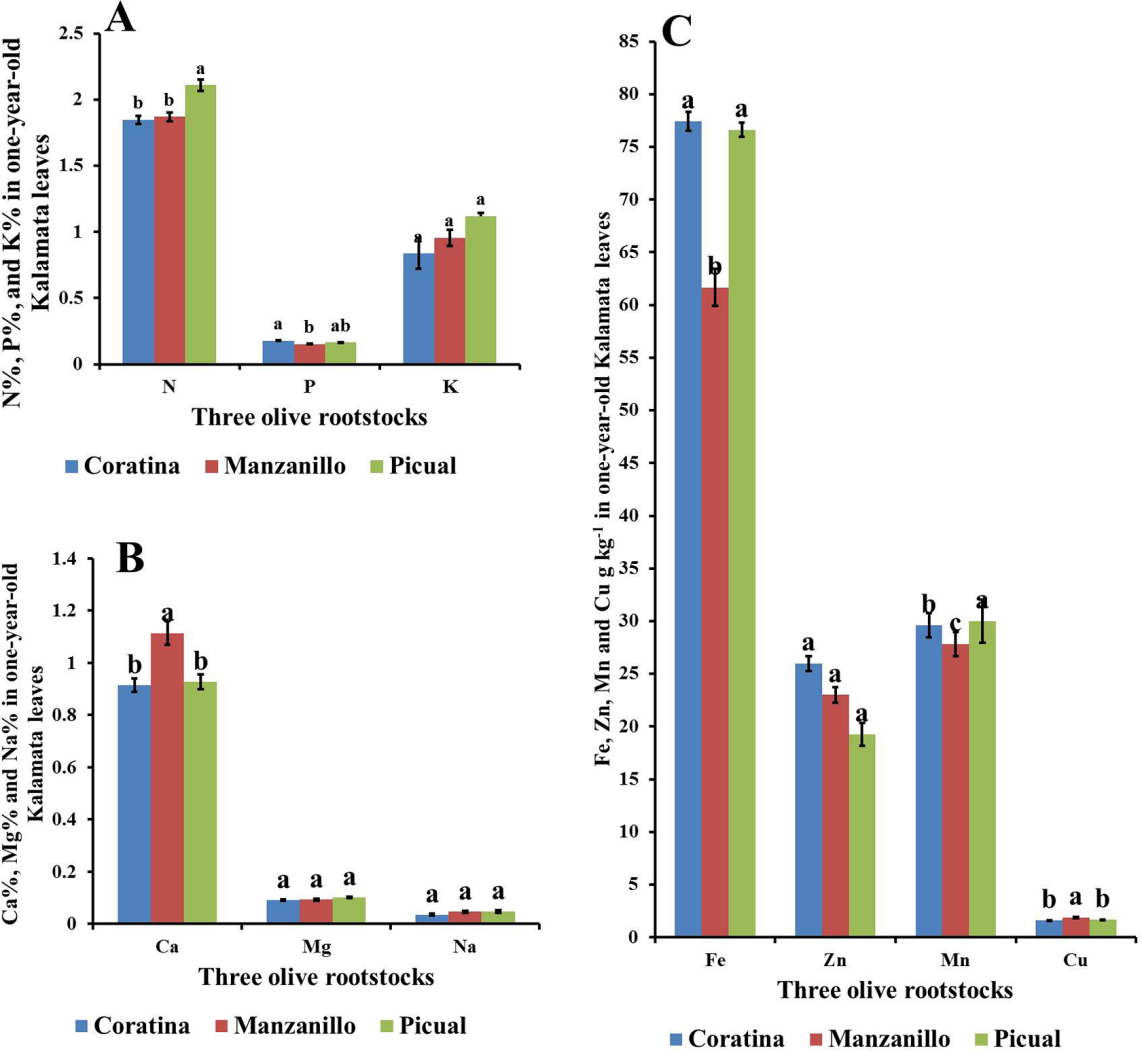
Effect of three olive rootstocks (Coratina, Manzanillo, Picual) on mineral content (**A**) (N, P, K), (**B**) (Ca, Mg, Na), and (**C**) (Fe, Mn, Zn, Cu) of one-year-old Kalamata plants. Data are mean ± standard error (n = 3). Different letters between treatments indicate significant differences (*p* < 0.05 level)

Picual rootstock significantly accumulated more N, Fe, Mn than Manzanillo rootstock in one-year-old Kalamata plants (Fig. 7A, C). While there is no differences between the rootstocks in K%, Mg%, Na% and Zn contents (Fig. B, C).

## Visual compatibility symptoms

**Fig. 8:**
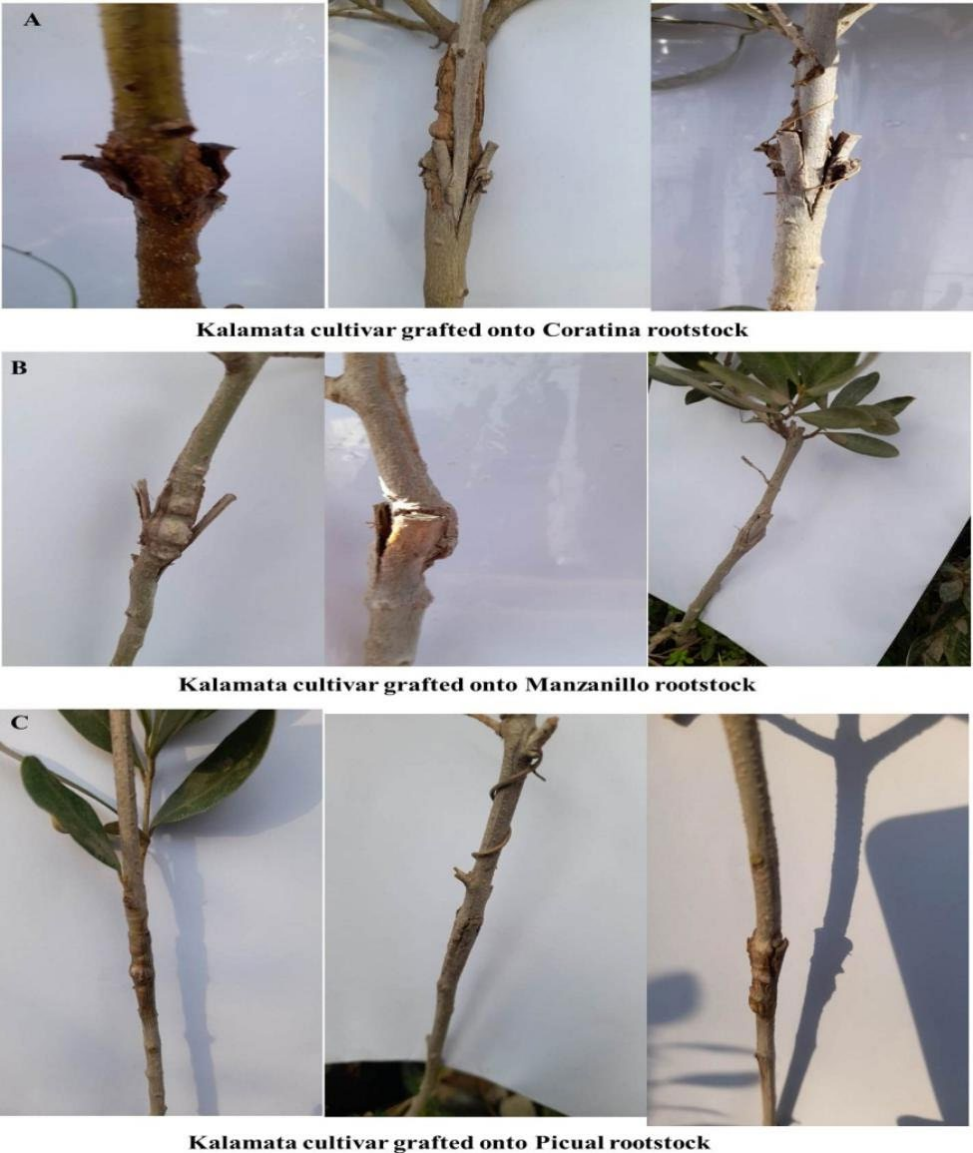
visual symptoms of compatibility in different graft combination in one-year-old Kalamata cultivar grafted onto three olive rootstock namely Coratina (**A**), Manzanillo (**B**) and Picual (**C**)

Visual examination of grafting union in one-year-old Kalamata plants grafted onto Coratina, Manzanillo and Picual rootsocks showed that there was a high degree of bark cracking in Kalamata/Manzanillo graft combinations followed by Kalamata/Coratina graft combinations, while Kalamata/Picual graft combination showed a good level of callusing and healing rootstock. Also, in Kalamata/Manzanillo graft combination there was an increase in union diameter beside cracking of the bark (Fig. 8A, B, C).

## Discussion

Formation of graft union is a complicated process [31]. Previous studies have demonstrated that plant grows more vigorously with a high level of graft compatibility [12]. The scion/rootstock combination of Flame seedless/Paulsen performed better than Flame seedless/Freedom, with vigorous growth and high photosynthetic capacity [12]. The results indicated that, Picual rootstock had the highest grafting success while Manzanillo rootstock had the lowest significant grafting success. These differences were attributed to the morphological and biochemical differences between the three olive rootstocks as described and discussed later. Preliminary analysis of leaves number and area showed that Kalamata plants grafted onto Picual rootstock compared to Coratina rootstock, while Manzanillo rootstock recorded the least significant leaves number and area (Fig. 1). These results were agreed with Moing et al. [32] who reported a significant reduction in incompatible peach (Batsch)/plum (Myrobolan P18) 55 days after grafting. Also, Arbequina cv. recorded the highest grafting success and leaves number, whereas Kalamata cv. had the lowest grafting success and leaves number when they grafted onto wild olive rootstock [9] (Hussain et al., 2016). Furthermore, visible symptoms of incompatibility showed that Manzanillo rootstock recorded a high profile of union cracking appearance (Fig. 8). In this regard, Azimi et al. [10] found slowly cambium cells formation, which had greater concentrations of TP in Ayvalik/Gemlik and Domat/Gemlik graft combinations than Sari Ulak/Gemlik and Memecik/Gemlik graft combinations.

Recent studies have reported that, SPAD reading which is an indicator of chlorophyll content or photosynthetic activity can be used as a compatibility indicator between scion and rootstocks [13, 33]. Studies have shown that the differences in the effects of different rootstocks on the scion behavior are mainly appeared in the leaves. Different rootstocks can induce significant differences in the scion chlorophyll content, which conversely affects photosynthetic rate [34]. In this regard, photosynthesis increased in the case of high compatible apple rootstock [35]. By contrast, Irisarri et al. [36] found in the incompatible pear (Williams)/quince (BA29) grafts combination abnormal shape of chloroplasts as well as poor developed membranes lacking grana. Also, the lower SPAD value may be accompanied by blockage in both nitrogen uptake and carbohydrate assimilates in scion leaves [37]. Our results indicated that, the highest compatible Kalamata/Picual followed by Kalamata/Coratina graft combinations produced more chlorophylls content as SPAD value as well as N leaf content.

Sugars consider one of the first products from phytosynthesis process. It play a simulative role in formation of graft union in several grafted plants such as cucumber/pumpkin [14], Flame seedless/Freedom [12] and Flame seedless/Paulsen [11]. This role of sugars is due to the regulation of rapamycin, which plays a vital role in controlling cell size, cell proliferation, transcription, autophagy, photosynthesis and metabolism of carbon and nitrogen [38, 39]. On the contrary, the results indicated that, the highest compatible Kalamat/Picual followed by Kalamata/Coratina rootstocks produced significantly lower sucrose and TS compared to Manzanillo rootstocks which accumulated more TS accompanied with lower significant sugar metabolism enzymes (AI and SCS) activity at the grafting union as well as the lowest leaf area and number of leaves in the scion. These findings may be due to the highest compatible rootstocks transferring sugars produced in the leaves directly for both graft partners (leaves and roots) which appeared in increasing leaf area and leaves number of their scion reverse to the lowest compatible Manzanillo rootstock. These results were consistent with Moing et al. [32]who found that, soluble sugars accumulated in the scion were coincided with grafting incompatibility, since rootstocks in more incompatible combinations resulted in more soluble sugar accumulation in the scion. More recently, Wang et al. [13] reported that the lowest survival rate was recorded in ‘Harumi’ as an interstock in apple grafting which accumulated higher sugars content and showed smaller leaf area and symptoms of incompatibility.

The wound response in grafting propagation generated reactive oxygen species, such as hydroxyl radicals, hydrogen peroxide and superoxide. Plants possess an antioxidant enzymatic system such as catalase and peroxidase for mitigating the adverse effects of these free radicals that can damage cell functions and structure [40]. Numerous studies have been declared that higher peroxidase activity in the grafting union was coincided with a lower graft-compatible combinations in grapevine [11, 12] and Prunus spp. [41]. Also, Zarrouk et al. [41] found high peroxidase activity in Peach (Summergrand) /Plum(Myrobalan GF 3–1) graft combination four to six months after grafting. Free radicals in the graft union consider a sign of graft incompatibility [36, 40].

Moreover, accumulation of TP may be related to incompatibility in several plants such as *Uapaca kirkiana* fruit [42], grapevine [11, 12] which restrict grafting process. According to Azimi et al. [10] grafted Ayvalik and Domat olive cultivars onto Gemlik rootstock formed cambium cells slowly, which had greater concentrations of TP compounds like Ferulic acid and 4-Hydroxyphenylacetic acid compared to Sari Ulak and Memecik cultivars. On the contrary, Telles et al. [43] reported that higher peroxidase activity and TP compounds have a positive effect on grafting union formation. Our results showed that, Manzanillo rootstock recorded the highest significant POX activity and TP content compared to the other two rootstocks. Moreover, Picual rootstock recorded the lowest CAT activity and TP content as well as POX activity compared to the other two rootstocks.

The role of phenolic compounds in grafting success depending on their concentration, time of accumulation, specific phenol type and plant species. The high concentration of phenolic compounds were observed in low compatible graft combination while the low concentration of phenolic compounds were observed in the highest compatible graft combination [10–12]. For the effect of specific phenolic compounds and plant species on grafting compatibility. higher concentrations of epicatechin and catechin were recorded in the incompatible cultivars of both apricot and quince [44, 45]. Also, catechin as well as procyanidin (B1, B2), and arbutin were recorded in incompatible pear tree [46]. Moreover, high concentration of gentisic acid, gallic acid, quercetin-3- glucoside, ellagic acid, catechin and p-coumaric acid have been recorded in low-compatible combinations of Eucalyptus gunnii [47]. On the other hand, sinapyl alcohol, *r*-coumaric alcohol and coniferyl alcohol are essential components for compatible graft combination [48]. For the accumulation time, in the low- compatibility graft combination, phenolic compounds were found in high concentration either 20 days after grafting [47], or 4 years after grafting [46]. But in the high- compatibility graft combination, it increased after grafting for lignification process then gradually decreased with increasing scion age. For the effect of Phenolic compounds on plant hormones, low-compatibility graft combination was associated with the accumulation of polyphenols at grafting union [49], which affect transportation of auxin [50]. In incompatible graft combinations, low IAA concentration may restrict phloem and xylem differentiation and lignification [51, 52]. Also, Phenols escape into the cytoplasmic matrix from the vacuole are oxidized by phenol oxidases and peroxidases [53]. Moreover, some of monophenol compounds are cofactors for IAA oxidase, while some of polyphenols compounds (chlorogenic, caffeic, protocatechuic and ferulic acids) inhibit IAA oxidation [54]. For the direct role of phenols on grafting compatibility, some of phenolic compounds (sinapyl alcohol, coniferyl alcohol and *r*-coumaric alcohol) has a positive effect on grafting compatibility [48], but in many cases it had many inhibitor effects. Phenolic compounds may be caused disruptions in the growth and development of xylem and phloem, which causing tissues necrotic in the grafting union [49, 55]. Also, polyphenol oxide activity in the incompatible graft combination was positively associated with dysfunctional in vascular connection and the cellular degeneration [41]. Moreover, phenols compound may cause lignin pathway inhibition and oxidized by phenol oxidases and peroxidases [53]. These previous studies were in agreement with our findings for either a currently grafted ‘Kalamata’ cultivar or a one-year ‘Kalamata’ cultivar. Since a higher TP was observed in the lowest compatible ‘Manzanillo’ rootstock, whereas a lower TP was observed in the highest compatible ‘Picual’ rootstock. Also, the highest TP ‘Manzanillo’ rootstock recorded the highest POX activity, while the lowest TP ‘Picual’ rootstock recorded the lowest POX activity.

For nutritional status, these experiment showed that, the less compatible Manzanillo rootstock had a lower nitrogen content compared to Coratina and Picual rootstocks. The ability of rootstock to increase nitrogen and magnesium in their scion could by more important characters due to save nitrogen fertilization and lower environment pollution which may be increased scion leaves and SPAD value in Picual and Coratina rootstocks. These compatibility results may be explain the previous results by Mofeed [8] who stated that, Kalamata cultivar grafted onto Picual rootstock recorded the highest increase in vegetative behavior (trunk diameter, tree height, shoot length) and fruiting characteristics (fruit set, yield) compared to Koroneiki and Manzanillo rootstocks which affected Kalamata growth and yield negatively. In this context, Laz [6] found that, Kalamata grafted into Picual rootstock exhibited more vegetative growth (number of leaves, leaf area) than Koroneiki rootstock under salinity stress. Moreover, the lowest Na and Cl content and the highest N% and K% were recorded in Frantoio and Koroneiki rootstocks. Also, Freedom rootstock had efficiency to accumulate more nitrogen content than Paulsen rootstocks in grapevine [7]. Also, leaf nitrogen content (% dry weight) fell in the incompatible graft combination peach(Batsch)/plum (Myrobolan P18) 65 days after grafting [32].

Our results indicated that, the most compatible Picual rootstock for Kalamata cultivar was accompanied with lower TP compounds and ABA content as well as higher GA_3_ content than the lowest compatible Manzanillo rootstock. In this regard, Su et al. [15] reported that ABA and tannin (phenolic compound) had negative effects on graft union formation in pecans (*Carya illinoinensis*). The results indicated that the lowest incompatible graft combination (Kalamata/Manzanillo) recorded the highest total indoles and IAA content compared to Kalamata/Picual the highest compatible graft combinations (Fig. 3). By contrast, Rasool et al. [34] mentioned that, auxins consider the regulation key in formation of graft union in horticultural plants. Also, auxin can modulate the vascular reconnection two days after grafting [31]. This difference may be explained by Aloni et al. [51, 56,] as he mentioned that, grafting incompatibility was accompanied with the auxin transport basipetallly into the rootstock where it stimulate oxidative stress and ethylene production. Moreover, endogenous IAA after wounding does not change significantly [57]. Moreover, Gainza et al. [58] showed an explanation for auxin mechanism in grafting incombatibility, since IAA was higher in incompatible graft combination than the compatible one. Where application of IAA transport inhibitor for incompatible graft combination negated root degradation. Moreover, the incompatible grafts showed normal shoot and root development after blocking IAA basal transport due to stem girdling [44]. Higher ABA was responsible for alter development of xylem and reduce of hydraulic conductance in dwarfing apple rootstocks [59]. Also, ABA can inhibit meristem activity, callus growth and wound-healing [58, 60].

Choosing the most compatible olive rootstock is a complex process. This study concluded that grafting compatibility was associated with morphological, physiological and biochemical changes in grafting union. The lest compatible Manzanillo rootstock displayed lower morphological (SPAD value, leaves number and leaf area) traits which was coincided with lower sugar enzyme activity (ACI, SCS) as well as higher TP, antioxidant enzyme activity (POX, CAT) in the grafting union. Further researches for extending the evaluation time of grafts combination were needed. Overall, ‘Picual was the most suitable rootstock for Kalamata olive cultivar. Finally further studies will be needed for determine specific sugars, specific phenol type as well as anatomical study for deep understand of grafting compatibility in olive cultivars.

## Data Availability

The authors included all generated and analyzed data in this article.

## Abbreviations

ABA: Abscisic acid hormone
ACI: acid Invertase enzyme
ANOVA: Analysis of variance
CAT: Catalase enzyme
cv.: Cultivar
GA: Gibberellic acid hormone
IAA: Indole actic acid hormone
POX: Peroxidase enzyme
SCS: Sucrose synthase enzyme
SPAD: Colorimeter indicator of chlorophyll content
TP: Total Phenols
TS: Total sugars.

## References

[1] IOC International Olive Council Available online. : http://www.internationaloliveoil.org, accessed on 30 December 2022.

[2] Tanasijevic L, Todorovic M, Pereira LS, Pizzigalli C, Lionello P (2014). Impacts of climate change on olive crop evapotranspiration and irrigation requirements in the Mediterranean region. Agric Water Manag.

[3] Trabelsi L, Gargouri K, Ayadi M, Mbadra C, Ben Nasr M, Ben Mbarek H, Ghrab M, Ben Ahmed G, Kammoun Y, Loukil E, Maktouf S, Khlifi M, Gargouri R (2022). Impact of drought and salinity on olive potential yield, oil and fruit qualities (cv. Chemlali) in an arid climate. Agric Water Manag.

[4] Wiesman Z, Lavee S (1995). Enhancement of stimulatory effects on rooting of olive cultivar stem cuttings. Sci Hort.

[5] Denaxa NK, Vemmos SN, Roussos PA (2021). Shoot Girdling improves rooting performance of Kalamata Olive Cuttings by Upregulating Carbohydrates, Polyamines and Phenolic Compounds. Agriculture.

[6] Laz SI (2004). Effect of irrigation with salinized water on growth and chemical constituents of “kala-mata” olive cultivar grafted onto different olive rootstocks. Arab Univ J Agric Sci Ain Shams Univ Cairo.

[7] Abdel-Mohsen MA, Rashdy AA (2015). Nitrogen and potassium uptake and utilization with four grapevine rootstocks. J Plant Production Mansoura Univ.

[8] Mofeed AS (2016). Effect of different Rootstocks on Flowering, Fruiting and Yield of Kalamata and Dolce Olive Trees. J Plant Production Mansoura Univ.

[9] Hussain I, Naeem N, Jan A, ur Rehman H, Ziaullah, Ali S (2016). Performance of different olive cultivars under time of grafting. Pure Appl Biol.

[10] Azimi M, Özkaya MT, Çölgecen H, Büyükkartal HN (2016). Analysis of phenolic compounds for determination of cambium differentiation and tracheal elements in olive graft combinations. J Exp Biol Agric Sci.

[11] Fayek MA, Rashedy AA, Ali AEM (2022). Alleviating the adverse effects of deficit irrigation in flame seedless grapevine via Paulsen interstock. Rev Bras Frutic.

[12] Fayek MA, Rashedy AA, Mahmoud RA, Ali AEM. Biochemical indicators related to grafting compatibility in Grapevine. Volume 8. RJPBCS; 2017. pp. 574–58. 3.

[13] Wang T, Deng L, Huang S, Xiong B, Ihtisham M, Zheng Z, Zheng W, Qin Z, Zhang M, Sun G, Wang J, Wang Z (2022). Genetic relationship, SPAD Reading, and Soluble Sugar Content as Indices for evaluating the graft compatibility of Citrus Interstocks. Biology.

[14] Miao L, Li Q, Sun T, Chai S, Wang CL, Bai LQ, Sun MT, Li YS, Qin X, Zhang ZH, Yu X (2021). Sugars promote graft union development in the heterograft of cucumber onto pumpkin. Hortic Res.

[15] Su WC, He HY, Liu ZZ, Mo ZH, Cao F, Peng FR. Physiological and biochemical changes during graft union formation in Carya illinoinensis. Biol Plant. 2021’65: 203–11.

[16] Habibi F, Liu T, Folta K, Sarkhosh A (2022). Physiological, biochemical, and molecular aspects of grafting in fruit trees. Hortic Res.

[17] Belaj A, Cipriani G, Testolin R, Rallo L, Trujillo I (2008). Characterization and identification of the main spanish and italian olive cultivars by simple-sequence-repeat markers. HortScience.

[18] Koubouris G, Bouranis D, Vogiatzis E, Nejad AR, GidayH, Tsaniklidis G, Ligoxigakis EK, Blazakis K (2018). Kalaitzis P and Fanourakis D. Leaf area estimation by considering leaf dimensions in olive tree. Sci Hortic.

[19] Van Handel E (1968). Direct microdetermination of sucrose. Anal Biochem.

[20] Miller GL (1959). Use of dinitrosalicylic acid reagent for determination of reducing sugar. Anal Chem.

[21] Larsen P, Harbo A, Klungron S, Ashein TA (1962). On the biosynthesis of some indole compounds in Acetobacter Xylinum. Physiol Plant.

[22] Unyayar S, Topcuoglu SF, Unyayar A. A modified method for extraction and identification of indole-3-acetic acid (IAA), gibberellic acid (GA3), abscisic acid (ABA) and zeatin produced by Phanerochaete chrysosporium, ME 446. Bulg J Plant Physiol; 1996: 22105–110.

[23] Sharma A, Shahzad B, Rehman A, Bhardwaj R, Landi M, Zheng B (2019). Response of phenylpropanoid pathway and the role of polyphenols in plants under abiotic stress. Molecules.

[24] Bradford MA (1976). Rapid and sensitive method for the quantitation of microgram quantities of protein utilizing the principle of proteindye binding. Anal Biochem.

[25] Aloni B, Pashkar T, Karni L (1991). Partitioning of [14 C]sucrose and acid invertase activity in reproductive organs of pepper plants in relation to their abscission under heat stress. Ann Bot.

[26] Aloni B, Karni L, Zaidman Z, Schaffer AA (1996). Changes of carbohydrates in pepper (Capsicum annuum L.) flowers in relation to their abscission under different shading regimes. Ann Bot.

[27] Lees R (1971). Laboratory Handbook of Methods of Food Analysis.

[28] Kacar B. Chemical Analysis of Soil and Plant. Ankara University Agri cultural Faculty Publication 1972;453. Ankara, Turkey: Ankara University (in Turkish).

[29] Freed R, Eisensmith SP, Goetz S, Reicosky D, Smail VM, Wollberg P. MSTAT-C A microcomputer program for the design, management and analysis of Agronomic Research Experiments. https://www.msu.edu/~freed/disks.htm., 1990.

[30] Snedecor GW, Cochran WG (1989). Statistical methods.

[31] Yin H, Yan B, Sun J, Jia P, Zhang Z, Yan X, Chai J, Ren Z, Zheng G, Liu H (2012). Graft-union development: a delicate process that involves cell–cell communication between scion and stock for local auxin accumulation. J Exp Bot.

[32] Moing A, Carbonne F, Gaudillère JP (1990). Growth and carbon partitioning in compatible and incompatible peach/plum grafts. Physiol. Plant.

[33] Wakiyama Y. The relationship between SPAD values and Leaf Blade Chlorophyll Content throughout the Rice Development Cycle Jpn Agric Res Q (JARQ). 2016;50, 329–34.

[34] Rasool A, Mansoor S, Bhat KM, Hassan GL, Baba TR, Alyemeni MN, Alsahli AA, El-Serehy HA, Paray BA, Ahmard P (2020). Mechanisms underlying graft union formation and rootstock scion interaction in horticultural plants. Front Plant Sci.

[35] Mao Y, Cui X, Wang H, Qin X, Liu Y, Hu Y, Chen X, Mao Z, Shen X (2022). Study of the grafting compatibility of the apple rootstock 12–2, resistant to apple replant diseases (ARD). BMC Plant Biol.

[36] Irisarri P, Binczycki P, Errea P, Martens HJ, Pina A (2015). Oxidative stress associated with rootstock-scion interactions in pear/quince combinations during early stages of graft development. - J Plant Physiol.

[37] Zarrouk O, Gogorcena Y, Moreno MA, Pinochet J. Graft compatibility between peach cultivars and prunus rootstocks. HortScience.2006;41,1389–94.

[38] Rodriguez M, Parola R, Andreola S, Pereyra C, Martínez-Noël G (2019). TOR and SnRK1 signaling pathways in plant response to abiotic stresses: do they always act according to the “yin-yang. model? Plant Sci.

[39] Jamsheer K, Jindal S, Laxmi A (2019). Evolution of TOR-SnRK dynamics in green plants and its integration with phytohormone signaling networks. J Exp Bot.

[40] Loupit G, Cookson SJ (2020). Identifying molecular markers of successful graft union formation and compatibility. Front. Plant Sci.

[41] Zarrouk O, Testillano PS, Risueño MDC, Moreno M, Gogorcena Y (2010). Changes in cell/tissue organization and peroxidase activity as markers for early detection of graft incompatibility in peach/plum combinations. J Am Soc Hortic Sci.

[42] Mng’omba SA, Du Toit ES, Akinnifesi FK, Venter HM (2007). Histological evaluation of early graft compatibility in Uapaca kirkiana Müell arg. scion/stock combinations. HortScience.

[43] Telles CA, Biasi LA, Mindêllo Neto UR, Deschamps C (2009). Fenóis totais, peroxidase e suas relações com a compatibilidade de mudas de pessegueiro interenxertadas. Cienc e Agrotecnologia.

[44] Musacchi S, Masia A, Fachinello J (2000). Variation of some enzymatic activities in relationship to scion/stock compatibility in pear/quince combinations. Acta Hort.

[45] Errea P, Garay L, Marin JA (2001). Early detection of graft incompatibility in apricot (Prunus armeniaca) using in vitro techniques. Physiol Plant.

[46] Hudina M, Orazem P, Jakopic J, Stampar F (2014). The phenolic content and its involvement in the graft incompatibility process of various pear rootstocks (Pyrus communis L). J Plant Physiol.

[47] De Cooman L, Everaert E, Curir P, Dolci M. The possible role of phenolics in incompatibility expression in Eucalyptus gunnii micrografts. Phytochem Anal. 1996; (7) 92–6.

[48] Bruce RJ, West CA (1989). Elicitation of lignin biosynthesis and isoperoxidase activity by pectic fragments in suspension cultures of castor bean. Plant Physiol.

[49] Feucht W (1992). The roles of phenolic compounds in graft incompatibility of prunus cerasus. Acta Hortic.

[50] Errea P (1998). Implications of phenolic compounds in graft incompatibility in fruit tree species. Sci Hortic.

[51] Aloni R, Cohen R, Karni L, Aktas H, Edelstein M (2010). Hormonal signaling in rootstock-scion interactions. Sci Hortic.

[52] Koepke T, Dhingra A (2013). Rootstock scion somatogenetic interactions in perennial composite plants. Plant Cell Rep.

[53] Hartmann HT, Kesler DE, Davies FT, Geneve RL (2002). Plant propagation. Principles and practices.

[54] Faust M (1989). Physiology of Temperate Zone Fruit Trees.

[55] Çölgeçen H, Azimi M (2015). Assessment of Graft compatibility of some Olive Cultivars on ‘Gemlik’ Rootstock by Florescence Microscopy. Jordan J Agricultural Sci.

[56] Aloni B, Karni L, Deventurero G, Levin Z, Cohen R, Katzir N, Lotan-Pompan M, Edelstein M, Aktas H, Turhan E, Joel DM, Horev C, Kapulnik Y (2008). Physiological and biochemical changes at the rootstock-scion interface in graft combinations between Cucurbita rootstocks and a melon scion. J Hortic Sci Biotechnol.

[57] Ikeuchi M, Iwase A, Rymen B, Lambolez A, Kojima M, Takebayashi Y, Heyman J, Watanabe S, Seo M, De Veylder L, Sakakibara H, Sugimoto K (2017). Wounding triggers callus formation via dynamic hormonal and transcriptional changes. Plant Physiol.

[58] Gainza F, Opazo I, Muñoz C. Graft incompatibility in plants: metabolic changes during formation and establishment of the rootstock/scion union with emphasis on Prunus species. Chil J Agric Res. 2015;75(Suppl1). 10.4067/S0718-58392015000300004.

[59] Tworkoski T, Fazio G (2015). Effects of size-controlling apple rootstocks on growth, abscisic acid, and hydraulic conductivity of scion of different vigor. Int J Fruit Sci.

[60] Zhou Q, Gao B, Li WF, Mao J, Yang SJ, Li W, Ma ZH, Zhao X, Chen BH (2020). Effects of exogenous growth regulators and bud picking on grafting of grapevine hard branches. Scientia Hort.

